# Vanity of Vanities

**Published:** 1895-11

**Authors:** 


VANITY OF VANITIES.—"Why should mortal man be proud?" I give it up. But that the average man is proud, in a sense of personal vanity, and the doctor is no exception to the rule,—seems very evident. Pride of character; pride of duty well done; pride of accomplishment of some noble end,—is pardonable; but *vanity*; vanity of personal appearance,—good looks—(and here comes in the question of good looks: a man who may be vain of his face or figure, and thinks himself handsome, may be, in my eyes or yours, quite the reverse) is unpardonable. A good looking face or a fine figure, is but an accident; and an accident, or a spell of sickness may destroy it. Why, then, be proud or vain of it? I do believe this species of vanity predominates in the genus *medicus*. The doctor, somehow, likes to look on his own face counterfeited on paper. There is a kind of fascination in it, which, to some, is irresistable. No matter, if that face, to another, have the expression of a frog, and be about as handsome; no matter, if the name be 'Grubs,' or 'Stubbs,' or "Tubbs," it looks pretty in print to the owner, and he loves to see it in the papers. Truly, a lowly ambition. We saw, recently, a reprint of some excellent clinics (St. Louis) illustrated by suitable cuts, and on the cover the face of the lecturer and operator; clearly an advertising dodge. But what kind of material can a medical journal editor's superior maxillary region be composed of; and what phase of vanity does he gratify; what *good*, what possible good can he expect to accomplish who prints his own lovely face in his own medical journal? The only possible explanation is that given by Squeers,—the Yorkshire schoolmaster,—for making Smike curry his horse; he made Smike spell 'horse' and then go and curry *his* horse to impress it upon his mind. Now, here is a medical editor who writes alleged smart things in his own journal. He follows it up by presenting in his editorial pages a half-tone engraving of his own lovely face, as much as to say, "Behold, *here* is the author of all those smart things! See! beneath this massive brow (and gold rimmed eyeglasses) a brain throbs as *is* a brain; a brain, capable of turning out editorials and paragraphs to astonish the natives; and not content with that, he affixes a string of "entitlements" to it a foot long—as if to say, "see—in recognition of my great intellect my fellow professors have dubbed me 'Professor' of ever so many things. Oh, but I'm some pumpkins! and do you not, in the press of business, nor in some unguarded moment, let it escape your memory."

